# Assessment of Carotid Arterial Stiffness in Community Settings With ARTSENS®

**DOI:** 10.1109/JTEHM.2020.3042386

**Published:** 2020-12-03

**Authors:** Jayaraj Joseph, P. M. Nabeel, Sudha Ramachandra Rao, Ramachandran Venkatachalam, Malay Ilesh Shah, Prabhdeep Kaur

**Affiliations:** 1 Department of Electrical EngineeringIndian Institute of Technology Madras 37268 Chennai600036India; 2 Healthcare Technology Innovation CentreIndian Institute of Technology Madras 37268 Chennai600113India; 3 Indian Council of Medical ResearchNational Institute of Epidemiology 29893 Chennai600077India

**Keywords:** Carotid artery stiffness, vascular stiffness, pulse wave velocity, ARTSENS, endothelial dysfunction, population study, ultrasound, carotid distensibility, population distribution

## Abstract

Objective: We investigate the field feasibility of carotid stiffness measurement using ARTSENS® Touch and report the first community-level data from India. Method: In an analytical cross-sectional survey among 1074 adults, we measured specific stiffness index ($\beta $), pressure-strain elastic modulus ($\text{E}_{\text {p}}$), arterial compliance (AC), and one-point pulse wave velocity (PWV$_{\beta }$) from the left common carotid artery. Data for established risk factors (waist circumference, blood pressure, plasma glucose, triglycerides, and HDL-C) were also collected. The association of carotid stiffness with age, gender, hypertension/diabetes, smoking, and clustering of risk factors was studied. Results: Measurements were repeatable with a relative difference (RD) between consecutive readings of < 5% for blood pressure and < 15% for $\sim 80$% of arterial diameter values. The average RDs for $\beta $, $\text{E}_{\text {p}}$, AC, and PWV$_{\beta }$, were 20.51%, 22.31%, 25.10%, and 14.13%, respectively. Typical range for stiffness indices among females and males were $\beta $: 8.12 ± 3.59 vs 6.51 ± 2.78, $\text{E}_{\text {p}}$: 113.24 ± 56.12 kPa vs 92.33 ± 40.65 kPa, PWV$_{\beta }$: 6.32 ± 1.38 ms^−1^ vs 5.81 ± 1.16 ms^−1^, and AC: 0.54 ± 0.36 mm^2^ kPa^−1^ vs 0.72 ± 0.38 mm^2^ kPa^−1^. Mean $\beta $, $\text{E}_{\text {p}}$, and PWV$_{\beta }$ increased (and mean AC decreased) across decades of age; the trend persisted even after excluding hypertensives and subjects with diabetes. The odds ratio of presence of multiple risk factors for $\text{E}_{\text {p}} \ge93.71$ kPa and/or PWV$_{\beta } \ge6.56$ ms^−1^ was ≥ 2.12 or above in males. In females, it was just above 2.00 for $\text{E}_{\text {p}} \ge91.21$ kPa and/or PWV$_{\beta } \ge5.10$ ms^−1^ and increased to ≥ 3.33 for $\text{E}_{\text {p}} \ge143.50$ kPa and ≥ 3.25 for PWV$_{\beta } \ge7.31$ ms^−1^. Conclusion: The study demonstrated the feasibility of carotid stiffness measurement in a community setting. A positive association between the risk factors and carotid artery stiffness provides evidence for the device’s use in resource-constrained settings. Clinical Impact: The device paves the way for epidemiological and clinical studies that are essential for establishing population-level nomograms for wide-spread use of carotid stiffness in clinical practice and field screening of ‘at-risk’ subjects.

## I. Introduction

Cardiovascular Diseases (CVD) is a leading cause of several fatal and nonfatal events, accounting for 31% of all deaths worldwide [1]. Of these deaths, ~ 35% (6.2 million) occur in middle age (30–69 years) cohort [2]. As the magnitude of CVD morbidity and mortality continues to accelerate, the pressing need for increased awareness and improved risk stratification is recognized globally. Early detection and timely intervention are efficient strategies to control and manage CVDs, thereby reducing its risk in causing adverse events.

Altered hemodynamics, loss of arterial elastic properties, and change in arterial wall dynamics result in a range of CVD-related abnormalities. Hence, early vascular health markers measured in terms of biomechanical properties of arteries offer a unique view of the underlying disease progression [3]. Measurement of the stiffness of common carotid artery (being a more accessible site to a central artery) has thus become a key marker used in epidemiological as well as interventional cardiovascular research [4]–[6]. Despite the strong scientific support [6] and mounting evidence establishing carotid stiffness as a predictor of future CVD risks and all-cause mortality [7]–[10], the need for ultrasound imaging devices supplemented by customized image analysis modules has prevented wide-spread adoption of this early vascular health marker. Besides, cost and logistic complexity limits field-level vascular stiffness studies in the general population. (The first large-scale study on carotid stiffness in the world was reported in 2017, performed using an ultrasound echo-tracking system on 900 subjects in a clinical setting [11].) In India, on the other hand, the use of imaging systems in out-of-hospital settings or operating it by a non-certified sonographer are legally restricted owing to stringent regulations concerning sex determination of the fetus and subsequent feticide [12]. Thus, epidemiological population data on carotid stiffness and its prevalence are limited in India.

Over the years, our group has developed and clinically validated an image-free alternative – ARTSENS® – for non-invasive assessment of vascular wall dynamics and stiffness. The system exploits A-mode ultrasound data with intelligent algorithms for automated measurement, thereby eliminating the need for imaging equipment/reference, expert operator, manual annotation, as well as offline analysis. A detailed discussion on the ARTSENS® technology can be found elsewhere [13] –[20].

**FIGURE 1.**
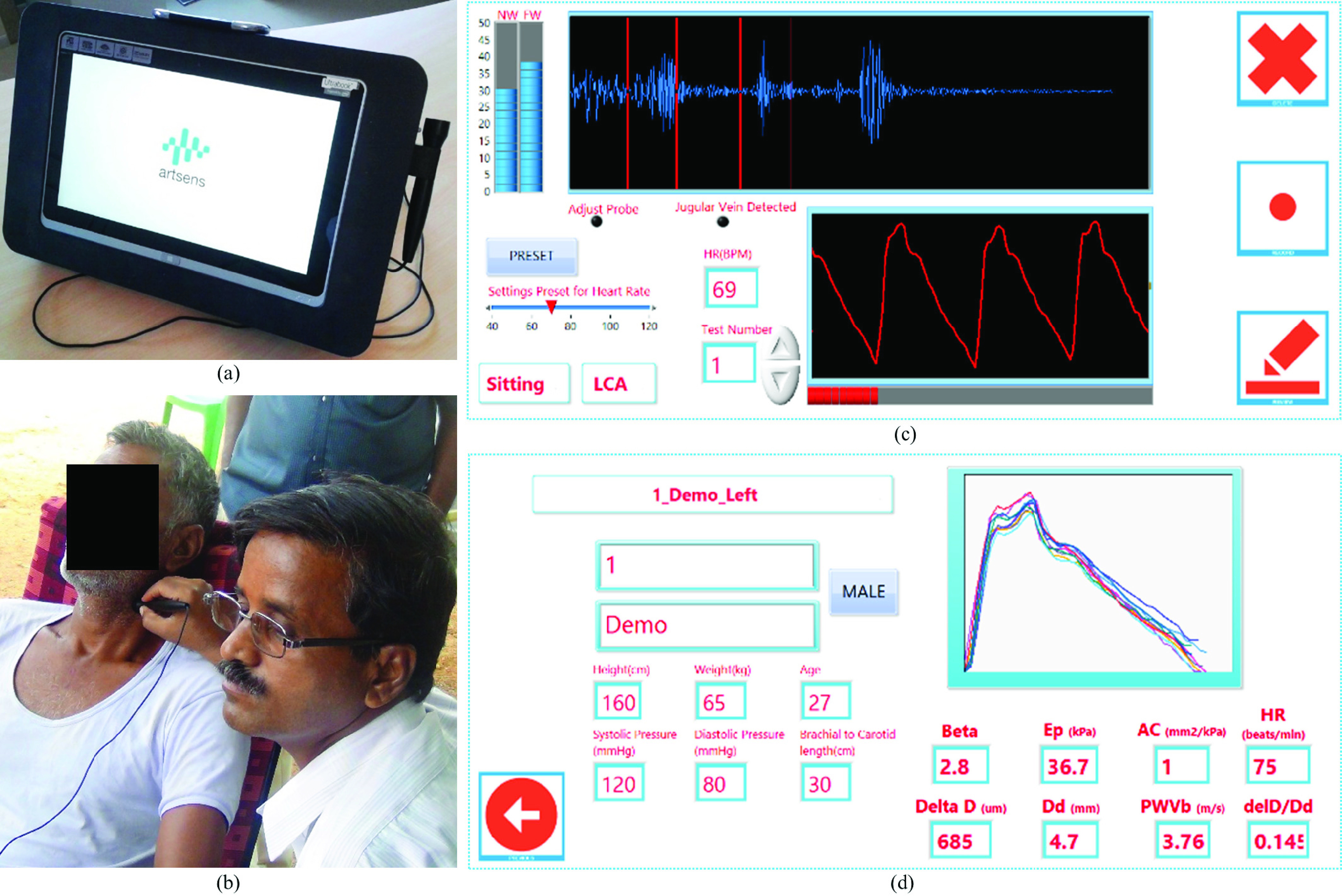
ARTSENS® Touch: (a) Device used in the field study for the measurement of carotid stiffness indices. (b) An operator performs stiffness measurement from the left common carotid artery of a subject. (c) Measurements page showing an echo frame and distension waveform. (d) Results page showing the recorded distension cycles and measured carotid stiffness indices.

ARTSENS® Touch (Fig. 1) is one of the most evolved forms of our image-free ultrasound technology vascular screening. It was developed as an easy-to-use, automated, field-deployable, and portable device for applications in the clinical and resource-constrained settings. It reduces the cost as well as skill barriers in using current imaging equipment. The device uses an image-free ultrasound probe (with a custom single-element transducer operated in pulse-echo mode) that the operator places over the common carotid artery, on the neck of the subject, yielding A-mode echoes along the scan axis. These continuous echoes are displayed in real-time to provide a visual (video-graphic) effect, which helps the operator precisely orienting the probe along the diameter of the artery by ensuring out-of-phase moving echoes from both the proximal and distal walls [14]. A set of automated algorithms identify and continuously track echoes originating from the walls and perform an online evaluation of the arterial lumen diameter and distension waveforms [14], [15], [17].

Beat-by-beat diameter parameters and blood pressure (BP) values are used to evaluate the measures of carotid artery stiffness [21], viz. specific stiffness index ($\beta$), pressure-strain elastic modulus ($\text{E}_{\mathrm {p}}$), arterial compliance (AC), and one-point pulse wave velocity (PWV$_{\beta }$). (Definitions and formulae of these stiffness indices are given in Supplemental Material.) Real-time measurement performed over consecutive cardiac cycles yields average stiffness indices and displays it for quick reference. The operator need not interact or provide manual inputs during the signal acquisition and measurement phase. Readers are advised to refer to Supplemental Material for a detailed understanding of the device’s specifications, in-built quality control measures, and operator training/performance evaluation.

In this work, we conducted the first community-based study in India to describe the distribution of carotid artery stiffness in a general population. Such an attempt has never been reported in large cohorts owing to the lack of appropriately usable, cost-effective, and field-deployable tools for epidemiological trails. This study aimed to (1) demonstrate the field deployability and measurement reliability of the ARTSENS® Touch in a resource-constrained setting, and (2) describe relationships between the carotid stiffness and cardiometabolic risk factors in an Indian population. The study methodology, observations, results, and the outlook are presented in the following sections.

## II. Materials and Methods

### A. Participants

The study executed as an analytical cross-sectional survey in a rural village of Tiruvallur district, Tamil Nadu, South India. The selected village is part of our institution’s field practice area; hence all the households were enumerated, and a line list was available for another ongoing cohort study. Since our primary objective was to investigate the distribution of carotid stiffness, and there exists a lack of community-level data from India to compute the sample size, all adults aged 30 years and above in the line list were considered eligible for the study. A total of 1074 subjects were recruited, and written informed consent was obtained prior to participation. Ethical approval was obtained from the National Institute of Epidemiology, Indian Council of Medical Research (NIE/IHEC/201407-02). The study was carried out in accordance with the latest revision of the Helsinki Declaration.

### B. Data Collection

All participants reported to our field setup as per preassigned slots. They were asked to fast for at least 10 hours and to abstain from dietary products known to alter stiffness (e.g. caffeinated beverages) for at least 18 hours prior to the study. Upon arrival, weight, height, waist circumference, and body-mass-index were measured. The data concerning risk factors such as age, tobacco use, alcohol consumption, current medication on hypertension and/or diabetes, and education were obtained using a structured questionnaire. Participants were asked to rest for at least 5 minutes on a comfortable chair with headrest.

All individuals underwent BP measurement on the right upper arm using an automated oscillometric apparatus (HEM-7101 – Omron, Japan), the measurement was repeated after 5 minutes and averaged for analyses. Blood sample (5 mL) was collected from the antecubital vein to perform laboratory tests, including high-density lipoprotein cholesterol (HDL-C), fasting plasma glucose (FPG), and triglycerides (TGL) serum level.

### C. Measurement Using Artsens® Touch

Carotid stiffness measurements were performed by either one of the two field operators who were trained at the beginning of the study period (Supplemental Material). Once the subject comfortably seated in a chair with back support, the operator recorded BP and entered it in the device. The operator then identified an approximate location of the left common carotid artery by palpation. The tip of the image-free ultrasound probe (applied with water-based ultrasound gel) was positioned near the identified location. The probe orientation was adjusted to get strong and sharp distinct echoes from both arterial walls as visually guided by the device and displayed on-screen (Supplemental Material). An on-screen progress bar fills up while capturing high-fidelity distension cycles, and the device automatically pops up results-page upon completing the measurement. All recorded data and results were saved into required file formats. Procedures were repeated after 10 – 15 minutes, and averaged values were used for analyses. The study photographs are given in Supplemental Material.

### D. Risk Factors

Clustering of three or more of the following cardiometabolic risk factors in an entity was considered as high-risk and marker of future events [22]. This definition otherwise characterizes the metabolic syndrome, which is associated with the structural and functional vascular abnormalities [22].

1) Central obesity: Waist circumference ≥90 cm in males or ≥80 cm in females (for Asian Indians).
2) Elevated TGL: TGL ≥150 mg/dL (1.7 mmol/L) or on drug treatment for elevated TGL.
3) Reduced HDL-C: HDL-C < 40 mg/dL (1.03 mmol/L) in males or < 50 mg/dL (1.29 mmol/L) in females, or on drug treatment for reduced HDL-C.
4) High BP: Systolic BP ≥130 mmHg or diastolic BP ≥80 mmHg, or on antihypertensive drug treatment.
5) Elevated FPG: FPG >100 mg/dL (5.6 mmol/L) or drug treatment for elevated FPG.

An individual who has smoked more than 100 cigarettes over his/her lifetime and continued to smoke at the time of the study (daily or occasionally) was considered as a current smoker. One who had quit smoking earlier was considered a former smoker [23]. An individual who had consumed alcohol in the past 12 months was reported as a current consumer. A regular consumer was identified as someone consumed alcohol one or more days per week. A former consumer defined as one who was drinking alcohol in the past but not in the last 12 months [24].

### E. Statistical Analysis

Results are given as mean ± standard deviation or minimum-to-maximum range, unless otherwise stated. Percentage relative difference (RD) of the measurements in 2 consecutive trials was obtained as an estimate of repeatability. It was calculated as the ratio of the absolute difference between successive readings to the absolute value of their mean. Gender-specific differences in the population distribution of stiffness indices were identified by subgroup analyses. Student t-test was performed to examine the equality of two means (male/female) for each of the stiffness indices. Analysis of variance was performed to compare means of stiffness indices in different age group (30 – 39, 40 – 49, 50 – 59, ≥60 years). Chi-square test was used to analyze the linear trend across the quintiles of stiffness indices and multiple risks. The relationship between the clustering of multiple (three or more) risk factors and ranges of each stiffness index (across quintiles) was examined separately for males and females. Logistic regression analysis was performed to compute unadjusted odds ratio (Model-1) and age-adjusted odds ratio (Model-2), with 95% confidence interval. Multiple regression analysis was used to identify the risk factors associated with stiffness indices and to develop predictive models. A p-value ≤0.05 was considered statistically significant. Analyses were conducted using PASW Statistics software (version 18.0, SPSS Inc., Chicago, USA).

## III. Results

### A. Measurement Feasibility in Out-Of-Hospital Setting

Among 1074 participants, reliable measurements (set of two) were performed on 983 (91.5%) as per protocol. Others’ data (8.5%) was excluded from the analysis due to any recent history of cardiac events or stroke (1.7%), the use of statins (0.8%), and dropout or incomplete measurements (6%) concerning practical challenges discussed in Section IV-A.

### B. Characteristics of the Study Population

Individual characteristics, for a comparison between male (N = 409, age = 46.8 ± 9.9 years) and female (N = 574, age = 46.9 ± 9.2 years) participants, are given in Table 1. Among the 983 subjects surveyed, 61% were 40 – 59 years of age and over one-fourth were illiterate. About 22% of the study population were farmers, and nearly half of females were homemakers. Smoking and/or alcohol consumption were prevalent among 44% and 55% males, respectively. About 45% of respondents reported moderate or vigorous physical activity. Among ~50% of the subject with high-risk level (≥3 metabolic abnormalities), central obesity was prevalent among 43%, more than half diagnosed with reduced HDL-C, one-third had elevated TGL, and ~60% had either hypertension or diabetes.

**TABLE 1:** Sociodemographic, Behavioral, and Risk Factors of the Study Population (N = 983)

Characteristics	Male (N = 409)			Female (N = 574)		
	N	%	Value	N	%	Value
Age group						
30 – 39 (Years)	123	30.1	35.7 ± 1.9	142	24.7	35.9 ± 2.1
40 – 49 (Years)	121	29.6	44.3 ± 2.7	230	40.1	44.6 ± 2.9
50 – 59 (Years)	112	27.4	54.1 ± 2.7	139	24.2	53.9 ± 2.6
≥60 (Years)	53	13.0	64.1 ± 3.1	63	11.0	64.4 ± 3.2
Education						
Illiterate	63	15.4		214	37.3	
Std. 1-8	195	47.7		278	48.4	
Std. 9 or above	151	37.0		82	14.3	
Occupation (Male = 401, Female = 572)						
Agriculture	115	28.7		99	17.3	
Employee	182	45.3		182	31.8	
Home maker	–	–		265	46.3	
Others	104	25.9		26	4.5	
Use of tobacco products						
Non-smoker	201	49.1		496	86.4	
Current smoker	127	31.1		–	–	
Former smoker	54	13.2		–	–	
Chewing	27	6.6		78	13.6	
Alcohol consumption						
Current consumer	227	55.5		–	–	
Former consumer	182	44.5		–	–	
Physical activity						
Sedentary	220	53.8		315	54.9	
Moderate	105	25.7		225	39.2	
Vigorous	84	20.5		34	5.9	
Risk markers/Subjects with identified risks						
Waist circumference (cm)	85.2 ± 11.5			79.7 ± 10.5		
Central obesity	137	33.5		287	50.0	
Systolic BP (mmHg)	132.8 ± 20.4			131.5 ± 21.3		
Diastolic BP (mmHg)	86.1 ± 12.5			82.8 ± 11.3		
High BP	288	70.4		335	58.4	
FPG (mg/dL)	117.8 ± 54.5			117.1 ± 50.2		
Elevated FPG	235	57.5		357	62.2	
TGL (mg/dL)	159.4 ± 86.2			137.4 ± 75.3		
Elevated TGL	174	42.6		185	32.2	
HDL-C (mg/dL)	44.3 ± 6.2			45.3 ± 7.0		
Reduced HDL-C	114	29.0		409	74.4	
Metabolic syndrome	168	42.7		322	58.5	

### C. Repeatability of Measurements in the Field

The average carotid artery lumen diameter and distension for the first and second trial by the same operator were (5.23 ± 1.48 mm, 0.36 ± 0.14 mm) vs (5.51 ± 1.27 mm, 0.35 ± 0.13 mm), respectively. Nearly 80% of the diameter measurements fell within an RD ≤15%. Both the systolic and diastolic BP values yielded a group average RD < 5% (pooled over all the study population), with < 4% values outside respective confidence intervals (mean ± 1.96 standard deviation).

The group average of stiffness indices pooled over all the subjects was $\beta $: 7.37 ± 3.12, $\text{E}_{\mathrm {p}}$: 102.82 ± 46.60 kPa, PWV$_{\beta }$: 6.06 ± 1.26 ms^−1^, and AC: 0.58 ± 0.28 mm^2^kPa^−1^. The field measurement of $\beta $, $\text{E}_{\mathrm {p}}$, PWV$_{\beta }$, and AC yielded group average RDs of 20.5%, 22.3%, 14.1%, and 25.1%, respectively. PWV$_{\beta }$ depicted the highest repeatability with a standard deviation of differences equal to 0.89 ms^−1^. The standard deviation of differences for $\beta $, $\text{E}_{\mathrm {p}}$, and AC were 1.96, 33.45 kPa, and 0.24 mm^2^kPa^−1^, respectively.

### D. Gender- and Age-Specific Trends in Stiffness Indices

**FIGURE 2.**
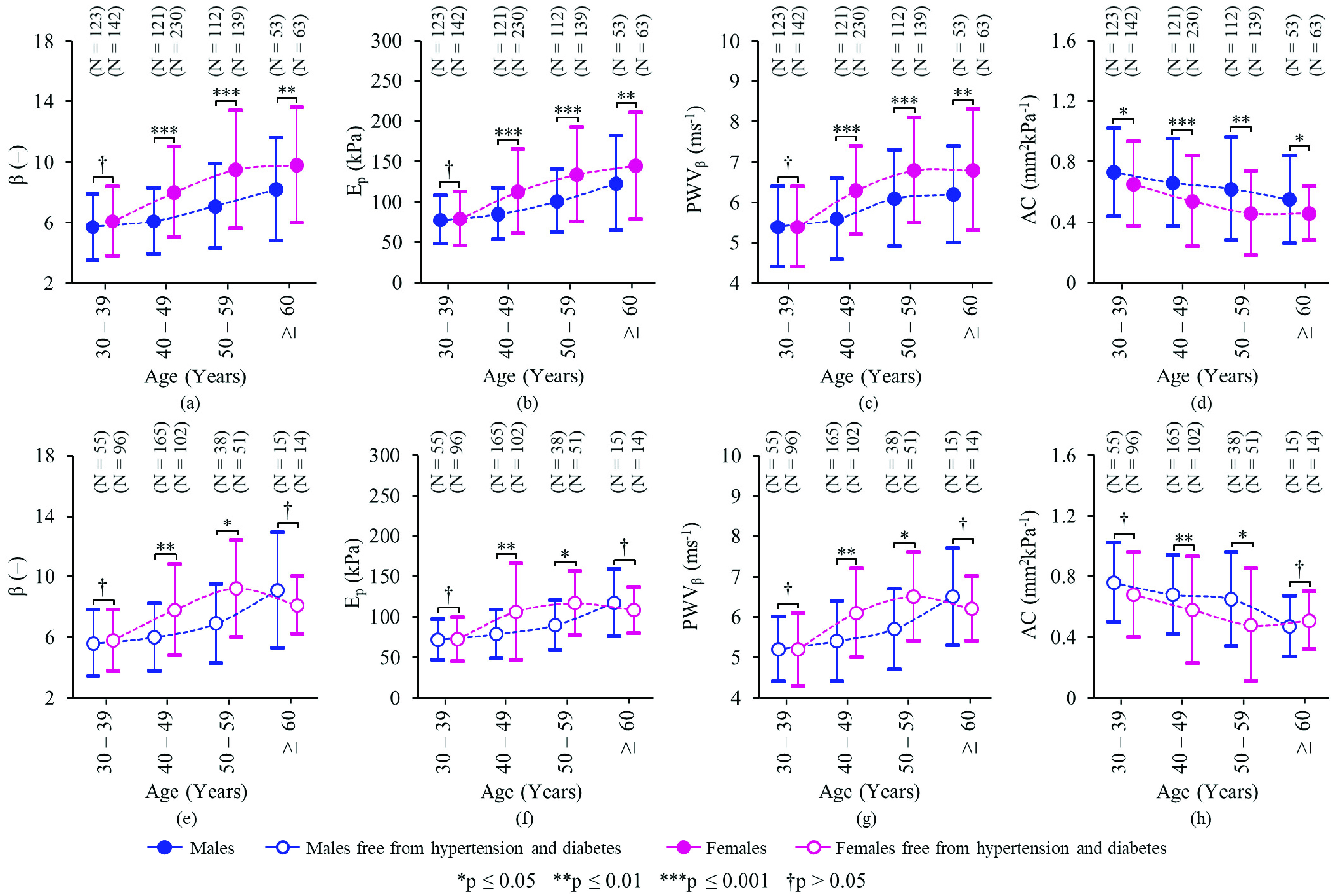
Gender-specific distribution of carotid stiffness indices (mean ± standard deviation of $\beta $, Ep, PWV$\beta $, and AC) across decades of age: (a – d) for the entire population (N = 983), and (e – h) for a subgroup excluding hypertensives and/or diabetes patients (N = 440).

The group averages of stiffness indices among females and males were $\beta $: 8.12 ± 3.59 vs 6.51 ± 2.78 (p < 0.001), $\text{E}_{\mathrm {p}}$: 113.24 ± 56.12 kPa vs 92.33 ± 40.65 kPa (p < 0.001), PWV$_{\beta }$: 6.32 ± 1.38 ms^−1^ vs 5.81 ± 1.16 ms^−1^ (p < 0.001), and AC: 0.54 ± 0.36 mm^2^kPa^−1^ vs 0.72 ± 0.38 mm^2^kPa^−1^ (p < 0.001). Fig. 2 depicts the gender-specific distribution of stiffness indices across decades of age for the study population (N = 983), as well as for a subgroup excluding hypertensives and/or subjects with diabetes (N = 440). Its descriptive statistics are given in Supplemental Material. As shown in Fig. 2(a)–(c) and Fig. 2(e)–(g), no significant difference was evident in the measures of $\beta $, $\text{E}_{\mathrm {p}}$, and PWV$_{\beta }$ for females and males belonging to the 30 – 39 years old cohort (p > 0.05). A significantly lower AC was observed for females of age 30 – 39 years old compared to males of the same group (p = 0.03; see Fig. 2(d)). However, this trend in AC was insignificant (p = 0.37) for the same age group free from hypertension and diabetes (Fig. 2(h)).

As in Fig. 2(a)–(c), females ≥40 years old presented with significantly higher mean values of $\beta $, $\text{E}_{\mathrm {p}}$, and PWV$_{\beta }$ than males of the corresponding age cohorts (p < 0.001 for 40 – 49 years and 50 – 59 years, and p < 0.01 for ≥60 years). Likewise, females aged 40 – 59 years old and free from both hypertension and diabetes showed significantly higher mean values of $\beta $, $\text{E}_{\mathrm {p}}$, and PWV$_{\beta }$ than those of males from same categories (Fig. 2(e)–(g); p < 0.01 for 40 – 49 years and p < 0.05 for 50 – 59 years). In turn, mean AC for females ≥40 years old was significantly lower than that of males from corresponding decade cohorts (Fig. 2(d)); p < 0.001 for 40 – 49 years, p < 0.01 for 50 – 59 years, and p < 0.05 for ≥60 years. A similar trend persisted even after excluding hypertensives and diabetes patients (Fig. 2(h)), which yielded p-values of < 0.01 and < 0.05 for 40 – 49 years and 50 – 59 years, respectively.

Note that the gradient in the mean of each stiffness index among males and females, across age groups (Fig. 2(a)–d)), increase from the younger (30 – 39 years) to the older (50 – 59 years) population, but became narrow for geriatric population (≥60 years). Differences in stiffness indices of geriatric females and males free from hypertension and diabetes (Fig. 2(e)–(h)) were statistically insignificant (p >0.05).

Regardless of gender, a significant increase (p < 0.001) in $\beta $, $\text{E}_{\mathrm {p}}$, and PWV$_{\beta }$ and a significant decline (p < 0.001) in AC were evident from the younger to older populations. The change in average stiffness ($\Delta$) between 30 – 39 years and 50 – 59 years were $\Delta \beta =2.49$ (42.3%, p < 0.0001), $\Delta \text{E}_{\mathrm {p}} =39.27$ kPa (49.7%, p < 0.0001), $\Delta $PWV$_{\beta } =1.05$ ms^−1^ (19.4%, p < 0.0001), and $\Delta $AC = −0.15 mm^2^kPa^−1^ (−21.9%, p < 0.0001). The corresponding measures for the subgroup free from hypertension and diabetes were $\Delta \beta =2.84$ (33.3%, p < 0.0001), $\Delta \text{E}_{\mathrm {p}} =37.35$ kPa (53.1%, p < 0.0001), $\Delta $PWV$_{\beta } =1.20$ ms^−1^ (23.5%, p < 0.0001), and $\Delta $AC = −0.12 mm^2^kPa^−1^ (−16.5%, p < 0.001). Age-specific variations in the carotid stiffness were apparent across successive decades ($\forall $ p < 0.01). However, no statistically significant differences were observed for stiffness indices of subjects ≥60 years old compared to their preceding age group ($\forall $ p >0.05).

### E. Association Between Multiple Risks and Stiffness Indices

The variation in stiffness of the carotid artery among subjects diagnosed with ≥3 risks and those with 0 – 2 risk(s) was studied separately for females (N = 550) and males (N = 393). (40 subjects were excluded due to unavailability of HDL-C data.) The prevalence of risk factors was 58.5% in female subjects and 42.7% in male subjects. Females with three or more risks exhibited significantly higher mean values of $\text{E}_{\mathrm {p}}$ and PWV$_{\beta }$ and an insignificant difference in mean values of $\beta $ and AC compared to those with less than three risks ($\beta $: 8.09 ± 3.09 vs 7.63 ± 2.91 (p = 0.08), $\text{E}_{\mathrm {p}}$: 116.38 ± 47.88 kPa vs 98.90 ± 41.49 kPa (p < 0.0001), PWV$_{\beta }$: 6.45 ± 1.25 ms^−1^ vs 5.98 ± 1.18 ms^−1^ (p < 0.0001), and AC: 0.52 ± 0.25 mm^2^kPa^−1^ vs 0.55 ± 0.26 mm^2^kPa^−1^ (p = 0.34)). Males exhibited a similar trend with the corresponding measures as $\beta $: 6.42 ± 2.53 vs 6.14 ± 2.12 (p = 0.23), $\text{E}_{\mathrm {p}}$: 93.59 ± 37.84 kPa vs 84.71 ± 31.05 kPa (p < 0.01), PWV$_{\beta }$: 5.86 ± 1.12 ms^−1^ vs 5.56 ± 1.02 ms^−1^ (p < 0.01), and AC: 0.67 ± 0.32 mm^2^kPa^−1^ vs 0.65 ± 0.29 mm^2^kPa^−1^ (p = 0.70). There was no difference in the proportions of multiple risks among males and females for $\beta $, $\text{E}_{\mathrm {p}}$, PWV$_{\beta }$, and AC ($\forall $ p >0.05). However, the proportions of multiple risks across the quintiles of $\text{E}_{\mathrm {p}}$ and PWV$_{\beta }$ was significant (p < 0.01). In contrast, that of $\beta $ and AC remained insignificant (p >0.05).

**FIGURE 3.**
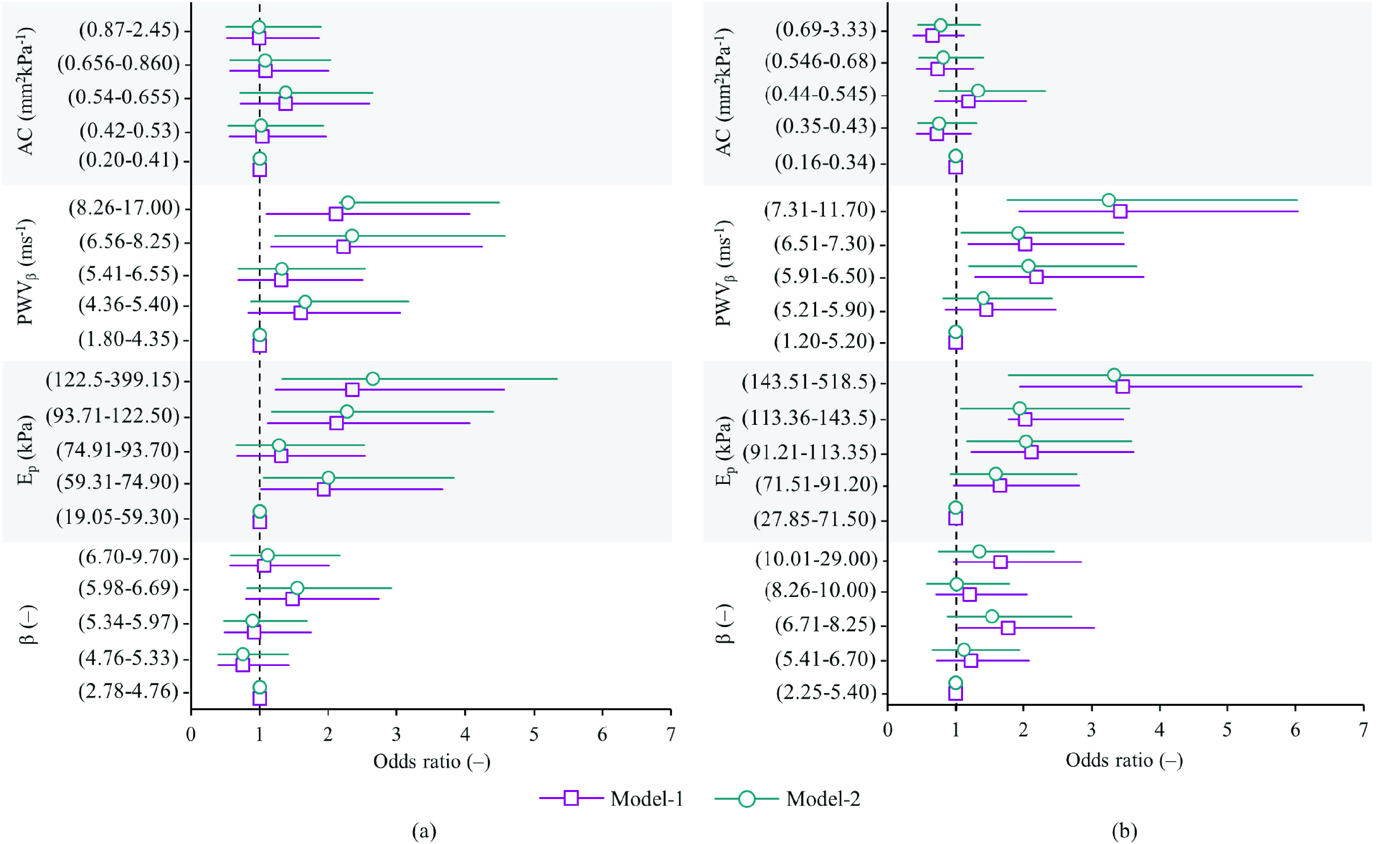
Odds ratio and 95% CIs for the clustering of multiple (three or more) cardiometabolic risk factors associated with carotid stiffness indices (across quintiles) among (a) male subjects (N = 393) and (b) female subjects (N = 550).

Logistic regression analysis yielded a significant relationship between the clustering of three or more risks and high values of $\text{E}_{\mathrm {p}}$ and PWV$_{\beta }$. Among males (Fig. 3(a)), the odds ratio of presence of multiple risk factors for $\text{E}_{\mathrm {p}} \ge $ 93.71 kPa and/or PWV$_{\beta } \ge6.56$ ms^−1^ (4^th^ and 5^th^ quintiles) was ≥ 2.12 or above in Model-1 as well as Model-2. Among females (Fig. 3(b)), the odds ratio for 91.21 kPa $\le ~\text{E}_{\mathrm {p}} \le $ 143.50 kPa and/or 5.91 ms^−1^ ≤ PWV$_{\beta } \le7.30$ ms^−1^ (3^rd^ and 4^th^ quintiles) was just above 2.00. The odds ratio increased to 3.45 (95% CI: 1.95 – 6.09) in Model-1 and 3.33 (1.78 – 6.25) in Model-2 for $\text{E}_{\mathrm {P}} \ge143.51$ kPa (5^th^ quintile), and 3.42 (1.94 – 6.03) in Model-1 and 3.25 (1.76 – 6.02) in Model-2 for PWV$_{\beta } \ge7.31$ ms^−1^ (5^th^ quintile). The highest odds ratio values for $\beta $ and AC were 1.55 (0.82 – 2.92) and 1.38 (0.72 – 2.65) in males (Fig. 3(a)) and 1.77 (1.03 – 3.04) and 1.33 (0.76 – 2.32) in females (Fig. 3(b)), respectively.

High BP was the most prevalent risk factor among the study population. Different hypertension stages [25] were diagnosed among 63.4% of the participants. 309 participants belong to the hypertension stage-1 group (systolic BP: 130 – 139 mmHg / diastolic BP: 80 – 89 mmHg), 296 to the hypertension stage-2 group (≥140/≥90 mmHg), and 18 to the hypertensive crises group (>180/>120 mmHg). There were 248 subjects with normal BP (<120/<80 mmHg) and 112 with elevated BP (120 – 129 / < 80 mmHg).

**FIGURE 4.**
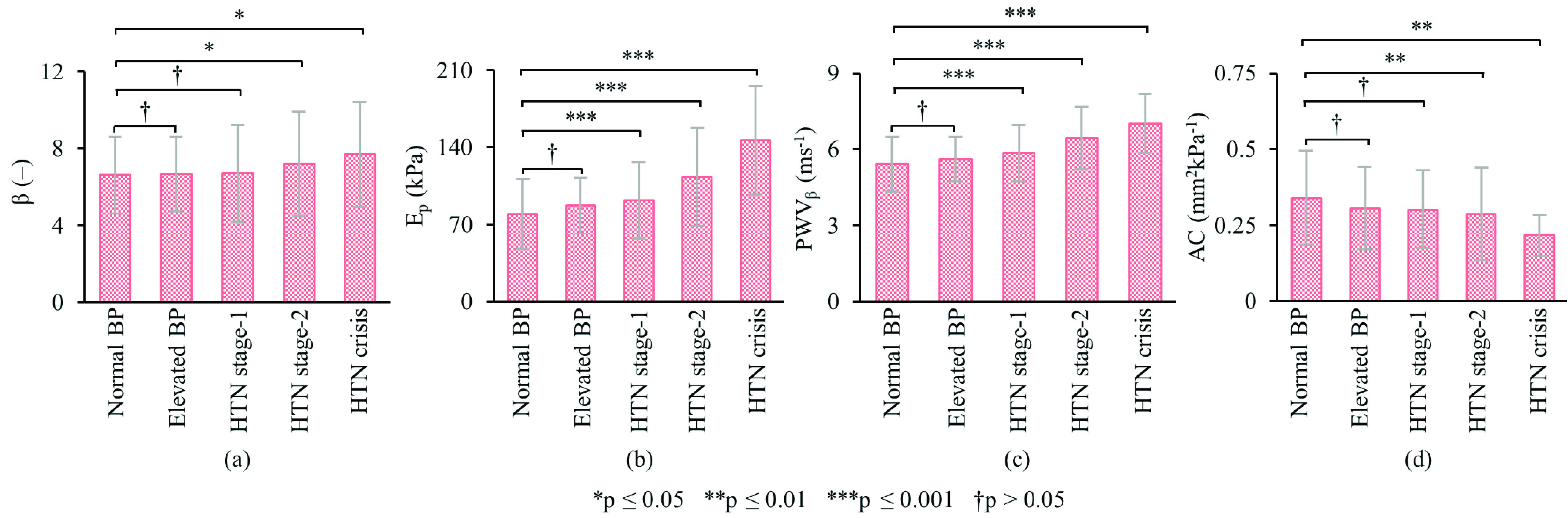
Mean carotid stiffness change, (a) $\beta $, (b) $\text{E}_{\mathrm {p}}$, (c) PWV$_{\beta }$, and (d) AC, by AHA/ACC BP categories. HTN = Hypertension.

As depicted in Fig. 4, the normal BP group possessed the lowest arterial stiffness (higher compliance) with average $\beta = 6.62\,\,\pm \,\,1.98$, $\text{E}_{\mathrm {p}} = 79.25\,\,\pm \,\,31.87$ kPa, PWV$_{\beta } = 5.41\,\,\pm \,\,1.08$ ms^−1^, and AC = 0.33 ± 0.16 mm^2^kPa^−1^. No significant difference was observed in $\beta $ (Fig. 4(a)), PWV$_{\beta }$ (Fig. 4(c)), or AC (Fig. 4(d)) of the normal versus elevated BP groups ($\forall $ p >0.05). The difference in their $\text{E}_{\mathrm {p}}$ (Fig. 4(b)) fell marginally short of statistical significance ($\Delta \text{E}_{\mathrm {p}} =7.67$ kPa, 9.7%, p = 0.057). The group averages of both $\text{E}_{\mathrm {p}}$ and PWV$_{\beta }$ were significantly higher in the hypertension stage-1 group compared to the normal BP group ($\Delta \text{E}_{\mathrm {p}} =12.38$ kPa, 15.6%, p < 0.001; and $\Delta $PWV$_{\beta } =0.44$ ms^−1^, 8.1%, p < 0.001). Conversely, the changes in $\beta $ and AC among hypertension stage-1 and normal BP participants were statistically insignificant ($\Delta \beta =0.06$, 0.9%, p >0.05; and $\Delta $AC = −0.02 mm^2^kPa^−1^, 6.1%, p >0.05). The values of $\text{E}_{\mathrm {p}}$ and PWV$_{\beta }$ showed an upsurge for subjects belonging to the hypertension stage-2 ($\Delta \text{E}_{\mathrm {p}} =33.82$ kPa, 42.7%, p < 0.001; and $\Delta $PWV$_{\beta } =1.03$ ms^−1^, 19.0%, p < 0.001) and hypertensive crises groups ($\Delta \text{E}_{\mathrm {p}} =66.78$ kPa, 84.3%, p < 0.001; and $\Delta $PWV$_{\beta } =1.59$ ms^−1^, 29.4%, p < 0.001). A significant rise in $\beta $ and decline in AC were also evident in hypertension stage-2 ($\Delta \beta =0.56$, 8.4%, p = 0.015; and $\Delta $AC _ −0.04 mm^2^kPa^−1^, −12.1%, p < 0.01) as well as in hypertensive crises subjects ($\Delta \beta =1.05$, 15.9%, p = 0.012; and $\Delta $AC = −0.11 mm^2^kPa^−1^, −33.3%, p < 0.01).

**FIGURE 5.**
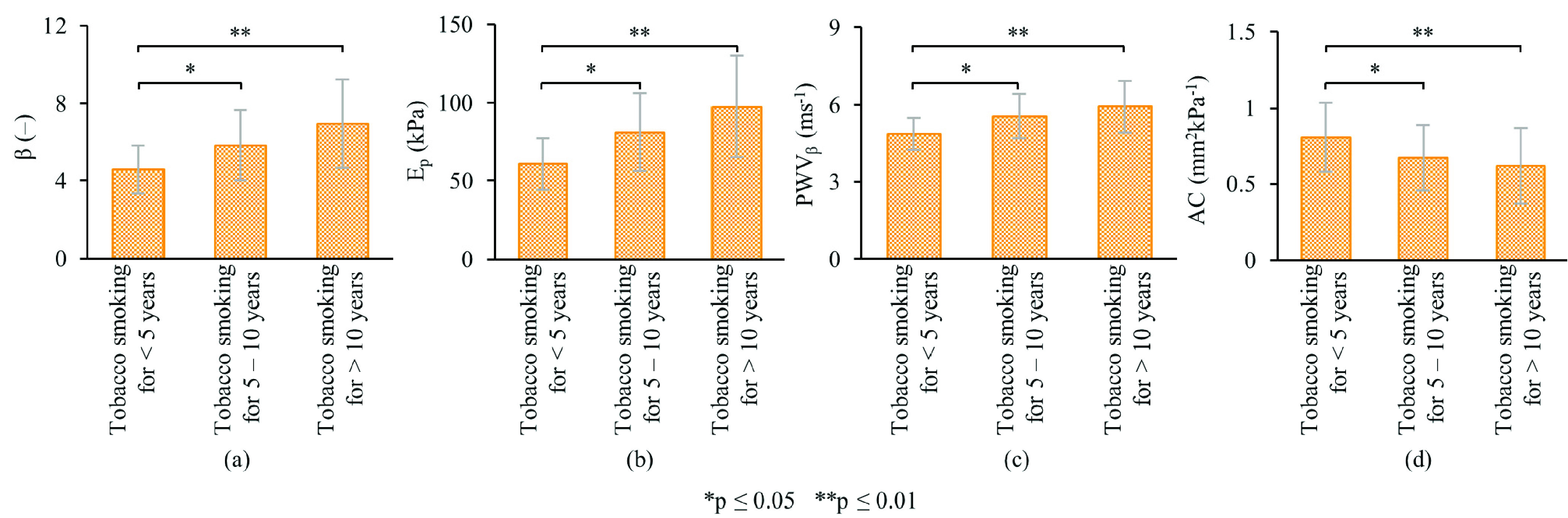
Mean carotid stiffness change, (a) $\beta $, (b) $\text{E}_{\mathrm {p}}$, (c) PWV$_{\beta }$, and (d) AC, by years of tobacco smoking.

Because gender was found to be an important determinant of arterial stiffening, the effect of smoking on the carotid stiffness was analyzed for (age-matched) males separately. (As given in Table 1, no female smokers available in the present study.) Although measured indices depicted a higher stiffness in current/former smokers than non-smokers ($\beta $: 6.41 ± 2.73 vs 6.79 ± 3.17; $\text{E}_{\mathrm {p}}$: 90.80 ± 39.93 kPa vs 95.38 ± 45.44 kPa; PWV$_{\beta }$: 5.65 ± 1.20 ms^−1^ vs 5.83 ± 1.36 ms^−1^; AC: 0.66 ± 0.33 mm^2^kPa^−1^ vs 0.62 ± 0.32 mm^2^kPa^−1^), their differences were marginally significant ($\forall $ p < 0.10). On the other hand, as depicted in Fig. 5, subjects who smoked for about 5 – 10 years showed significantly higher stiffness compared to those who smoked less than 5 years ($\Delta \beta =1.26$, 27.5%, p < 0.05; $\Delta \text{E}_{\mathrm {p}} =20.4$ kPa, 33.6%, p < 0.05; $\Delta $PWV$_{\mathrm {\beta }} =0.69$ ms^−1^, 14.3%, p < 0.05); and $\Delta $AC = −0.14 mm^2^kPa^−1^, −17.1%, p < 0.05). Compared with smokers of less than 5 years, the greatest changes in stiffness indices were seen in chronic smokers of more than 10 years ($\Delta \beta =2.38$, 51.9%, p < 0.01; $\Delta \text{E}_{\mathrm {p}} =36.72$ kPa, 60.4%, p < 0.01; $\Delta $PWV$_{\beta } =1.07$ ms^−1^, 22.0%, p < 0.01); and $\Delta $AC = −0.19 mm^2^kPa^−1^, −23.4%, p < 0.01).

Multiple regression analysis further demonstrated significant association (p < 0.01) between the stiffness indices and age ($\text{Y}_{\mathrm {AGE}}$), gender ($\text{V}_{\mathrm {SEX}}$), systolic BP ($\text{P}_{\mathrm {SYS}}$), diastolic ($\text{P}_{\mathrm {DIA}}$), number of clustered cardiometabolic risks ($\text{N}_{\mathrm {CMR}}$) and number of years of chronic smoking ($\text{Y}_{\mathrm {SMK}}$). The analysis also yielded predictive models that relate $\beta $, $\text{E}_{\mathrm {p}}$, PWV$_{\beta }$, and AC to measures of various significant risk factors, as given below:

\begin{align*} \mathrm {\beta }=&{0.087 }\mathrm {Y}_{\mathrm {AGE}}+\mathrm {1.361}{\mathrm { V}}_{\mathrm {SEX}}+\mathrm {0.046}{\mathrm { P}}_{\mathrm {SYS}} \\&-\,\mathrm {0.076}{\mathrm { P}}_{\mathrm {DIA}}+\mathrm {0.035 }\mathrm {Y}_{\mathrm {SMK}}+\mathrm {0.124 }\mathrm {N}_{\mathrm {CMR}}+2.099 \\ \mathrm {E}_{\mathrm {p}}=&\mathrm {1.021 }\mathrm {Y}_{\mathrm {AGE}}+\mathrm {22.182}{\mathrm { V}}_{\mathrm {SEX}}+\mathrm {1.177}{\mathrm { P}}_{\mathrm {SYS}} \\&-\,\mathrm {0.588}{\mathrm { P}}_{\mathrm {DIA}}+\mathrm {0.088 }\mathrm {Y}_{\mathrm {SMK}}+\mathrm {0.668 }\mathrm {N}_{\mathrm {CMR}}-73.246 \\&\hspace {-2pc} {\mathrm {PWV}}_{\beta } \\=&\mathrm {0.034 }\mathrm {Y}_{\mathrm {AGE}}+\mathrm {0.502}{\mathrm { V}}_{\mathrm {SEX}}+\mathrm {0.014}{\mathrm { P}}_{\mathrm {SYS}} \\&+\,\mathrm {0.067}{\mathrm { P}}_{\mathrm {DIA}}+\mathrm {0.012 }\mathrm {Y}_{\mathrm {SMK}}+\mathrm {0.074 }\mathrm {N}_{\mathrm {CMR}}+1.426 \\ \mathrm {AC}=&{1.251-0.003 }\mathrm {Y}_{\mathrm {AGE}}-\mathrm {0.124}{\mathrm { V}}_{\mathrm {SEX}}+\mathrm {0.006}{\mathrm { P}}_{\mathrm {SYS}} \\&+\,\mathrm {0.005}{\mathrm { P}}_{\mathrm {DIA}}-\mathrm {0.002 }\mathrm {Y}_{\mathrm {SMK}}+\mathrm {0.011 }\mathrm {N}_{\mathrm {CMR}}\end{align*}

Here, the value for gender was introduced into the equations as $\text{V}_{\mathrm {SEX}} =1$ for ‘male’ and $\text{V}_{\mathrm {SEX}} =2$ for ‘female’. Both $\text{Y}_{\mathrm {AGE}}$ and $\text{Y}_{\mathrm {SMK}}$ were in years; $\text{P}_{\mathrm {SYS}}$ and $\text{P}_{\mathrm {DIA}}$ were in units of mmHg; and the value for $\text{N}_{\mathrm {CMR}} \in $ {0, 1, 2, 3, 4, 5}. These models explained ~65% variability in each stiffness index (p < 0.001).

## IV. Discussion

### A. Measurement Feasibility and Challenges in the Field

ARTSENS® Touch, is a portable device with integrated modules (weight ≈ 2 kg), enabling easy handling in the field. All the measurements were performed without failures throughout the study. Device’s icon-based custom touch-screen GUI was appreciated for improved usability. A rugged enclosure (machined nylon) offered protection from mechanical impacts during transport. As no electrical outlet sockets were available in the field, the device was operated on its 3000 mAh internal battery. A fully charged device was sufficient for a day’s study, which lasted for nearly four hours.

Two sets of carotid stiffness measurement completed on 983 subjects demonstrate field usability of the device. The stiffness indices exhibited sufficient repeatability and are comparable to those reported in clinical studies conducted using ultrasound imaging systems [26]. Note that individuals with no technical or clinical background could be trained to operate ARTSENS® devices. Nevertheless, some practical challenges reported in this study should be underlined. Measurements could not be completed on a few geriatric subjects who had respiratory difficulty. Repeatability of the readings was compromised for a few morbidly obese subjects with fat deposition in the neck. Many participants expressed apprehension to the application of ultrasound gel on the neck. Explanations were provided to alleviate their concerns, which could be attributed to low education and health awareness.

Based on the study feedback, we have evolved the design and developed a much smaller device (ARTSENS® Pen) that takes power from a USB and operate with Microsoft Windows-based computer/tablet. This hand-held device (weight ≈ 100 g) with certified ingress protection greatly improves the portability and field-deployability. We have also integrated a custom solid ultrasound couplant into the updated probe for future studies.

### B. Carotid Stiffness Distribution in the Study Population

A gender-specific difference and an age-specific progression of the carotid stiffness were observed in the recruited subjects (Fig. 2). The observations were consistent in the subgroup free from both hypertension and diabetes. Female participants have shown marginally higher stiffness than males across all age groups above 40 years. This pattern of elevated arterial stiffness in older female participants is expected, and agrees with studies exploring gender modulated associations between vascular dysfunction and diseases progression [27]–[29]. Note that the progression of age-related stiffening of human arteries follows different patterns in males and females [27]–[29], with hormonal mechanisms having a substantial role in artery stiffening in females [50], [51]. Females in the postmenopausal period are at a higher risk of loss of endothelial function resulting in arterial stiffening [30], which was reaffirmed in the current study (Fig. 2). The study findings and measurement capabilities of the device potentially provide added insights to ongoing research on the relationship between gender and the progression of vascular stiffness with respect to age.

A nonlinear trend was evident in the age-specific progression of the carotid stiffness. All the stiffness indices accelerated from younger to older age-group, and later slowed in the geriatric subjects (Fig. 2). This pattern was comparable in both male and female groups with a higher progression rate in females. The observed age-trends of stiffness indices are in agreement with a previous large-scale study (conducted outside India) [31]. A few studies from various countries have also reported similar results describing the distribution of carotid stiffness and age-trends in limited samples [7], [11], [32]. Since the present study is the first to report values of carotid stiffness in an Indian population, a direct inter-study comparison of mean stiffness values cannot be strictly performed due to ethnic differences.

Recently, efforts have made to develop age- and gender-wise normative reference for carotid stiffness indices [31], [33]. Due to the lack of a database which was large enough, an attempt was also made to integrate multiple datasets from various cohort studies [33]. Such nomograms might be skewed due to the ethnic difference of study populations. Since ARTSENS® Touch has established its usability in resource-constrained settings, it can be reliably implemented in various sectors of primary healthcare chain for multi-centric data collection. This would help in establishing population-specific reference ranges and normative data for the carotid arterial stiffness.

### C. Potential of Carotid Stiffness for Risk Assessment

Strong positive associations of $\text{E}_{\mathrm {p}}$ and PWV$_{\beta }$ with established risk factors were observed. Ep varied over a wide range in the current population (28 kPa – 518 kPa). Since $\text{E}_{\mathrm {p}}$ is a direct measure of the vessel wall elasticity, it showed higher sensitivity to track changes in the stiffness caused by the presence of multiple risk factors. It has also been shown to be superior to all other indices in tracking the changes in carotid stiffness across BP categories. PWV$_{\beta }$, on the other hand, was the most repeatable measure of the carotid stiffness. It inherently accounts for arterial material properties and nonlinear characteristics of transmural pressure and lumen diameter interaction [5]. Note that the equation for PWV$_{\mathrm {\beta }}$ (Supplemental Material) is tolerant of error propagation. Therefore, it may be used as the most reliable and repeatable measure of vascular stiffness with applications in the field and high-throughput clinical studies.

It may be noted that distensibility coefficient (DC) is a well-studied marker of arterial stiffness, and has been reported to be an independent predictor of cardiovascular events and all-cause mortality [7], [9], [34]. By definition, DC is closely related to $\text{E}_{\mathrm {p}}$ and PWV$_{\beta }$ as; $\mathrm {DC \propto }\mathrm {E}_{\mathrm {p}}^{-1}$ and $\mathrm {DC \propto }{\mathrm {PWV}}_{\beta }^{-2}$ [35]. These measures obtained in the study were scaled to DC, and yielded significant association with established risk factors. The trends were consistent with previous studies [7], [9], [34]. On a similar note, while the agreement between carotid stiffness indices and gold-standard aortic stiffness have been moderate [36], PWV$_{\beta }$ yields better correlation [37]. Thus, it offers a potential way to directly compare the carotid arterial stiffness level with the aortic stiffness. Beyond the risk stratification, recent studies have identified PWV$_{\beta }$ as a strong predictor of left ventricular dysfunction [38] and coronary artery diseases [39]. Advanced pathophysiological applications of the PWV$_{\beta }$ above and beyond other *in-vivo* stiffness markers are summarized elsewhere [5].

Evaluation of AC is most prone to be corrupted by the error propagation as per the theoretical definition. Hence, it is least suited for use in field trials where the measurement repeatability cannot be as high as that in clinical settings. The measure of $\beta $ possesses a significant pressure-dependent bias when pooled over the population data [40]. Although it could be used for a quick comparison of population-specific results, studies have advised correcting the absolute $\beta $ in terms of a standard pressure to deduce ‘pressure-independent’ metric of vascular stiffness [40]. This will improve population-specific results on the distribution of risk factors/markers. It is worth noting that the current population had a high prevalence of hypertension and related alterations in their stiffness, which confound the relationship between $\beta $ and risk factors. The lack of association of $\beta $ with the studied risk factors tends to confirm considerations described above and shows its limitation when it comes to a population level large-scale vascular screening.

Finally, the observed high prevalence of multiple risk factors in the study population agrees with a previous community study performed in South India [41]. Another study from a similar cohort has reported increased vascular stiffness among people with diabetes [42]. The observed trend of an increased stiffness level in hypertensives and chronic smokers was also consistent with previous similar studies [43], [44]. Asian Indians indeed have a high risk of adverse cardiac events as compared to other ethnic groups due to the increased burden of multiple risk factors [45]. However, given the practical difficulties in stiffness evaluation, there is a lack of data on vascular stiffness and cardiovascular events in India. The present study, despite a cross-sectional one, revealed the association of carotid stiffness indices with the age, gender, and clustering of multiple risks.

Further cohort studies using our device would examine whether the excess CVD events associated with clustering of cardiometabolic risk factors are partly mediated through the amplified alterations in vascular properties and endothelial dysfunction. Note also that predictive models reported herein may be attributed to the characteristics of the population under study. A multiethnic cohort study to develop generalized models is worthy for future work.

### D. Strengths and Limitations of the Study

The major strength of this study is the use of a portable device to assess the carotid arterial stiffness in a resource-constrained field setting. The study demonstrated that the device enabled a reliable measurement of clinically accepted stiffness indices at a population-level. Thus, the developed device could overcome the limitations of ultrasound imaging equipment and operator expertise needed for stiffness evaluation. It also eliminates the need to access the groin region (which is required for gold-standard carotid-to-femoral PWV *viz.* aortic PWV), thereby making it an excellent choice for large-scale assessment of the vascular stiffness.

A major study limitation was the high prevalence of one or more cardiometabolic risk factors in the population. Therefore, we did not attempt to develop a nomogram for carotid stiffness indices for the current study. The lack of an existing nomogram (for Indian population) caused dissatisfaction in participants since it was impractical to provide a precise diagnosis of their vascular health after the test by comparing their results with that of nomogram values. The study had an inherent limitation in determining the temporal relationships of carotid stiffness with risk factors due to its cross-sectional nature. In general, only association and not causality can be inferred from the data. Selection of a specific region further restricted the extension of findings to the Indian population.

## V. Conclusion

We conducted the first community-level study in an Indian rural cohort to describe the distribution of the carotid stiffness. This study established a reliable assessment of carotid stiffness indices in out-of-hospital settings using ARTSENS® Touch. It demonstrated the functionality and sufficient repeatability for potential use in population-level screening. The age-/gender-specific distribution of stiffness indices was explored. A strong relationship between the stiffness indices and cardiometabolic risk factors indicates their utility for screening patients at high-risk in clinics as well as field settings. Learnings from this study were incorporated into the instrument hardware, probe design, and algorithms to develop smaller and easy-to-use prototypes. ARTSENS® devices enable large-scale field studies to develop population-specific nomograms, thereby establishing the innate role of carotid stiffness in cardiovascular risk stratification.

## Appendix

Supplementary Material (online) accompanies this paper.
